# Care of the Eyes in the Use of the Microscope

**Published:** 1863-09

**Authors:** 


Care of the Eyes in the use of the Microscope.—The *Westminster Review* gives the following instructive remarks upon this subject from the valuable work on "The Microscope and its Revelations," by Dr. Carpenter. "Although most miscroscopists acquire a habit of employing only *one* eye, (generally the right), yet it will be decidedly advantageous to the beginner that he should learn to use either eye indifferently; since by employing and resting each alternately, he may work much longer, without incurring unpleasant or injurious fatigue, than when he always employs the same. Whether or not he do this, he will find it of great importance to acquire the habit of *keeping open the unemployed eye.* This, to such as are unaccustomed to it, seems at first very embarrassing, on account of the interference with the microscopic image which is occasioned by the picture of surrounding objects formed upon the retina of the second eye; but the habit of restricting the attention to that impression only which is received through the microscopic eye may generally be soon acquired, and when it has once been formed, all difficulty ceases. Those who find it unusually difficult to acquire this habit, may do well to learn it in the first instance with the assistance of the shade just described, the employment of which will permit the second eye to be kept open without any confusion. The advantage of the practice, in diminishing the fatigue of long continued observation, is such that no pains are ill-bestowed by the microscopists which are devoted to early habituation to it. There can be no doubt that the habitual use of the microscope for many hours together, especially by lamplight, and with high magnifying powers, has a great tendency to injure the sight. Every microscopist who thus occupies himself, therefore, will do well, as he values his eyes, not merely to adopt the various precautionary measures already specified, but rigorously to observe the simple rule of *not continuing to observe any longer than he can do so without fatigue.*"—*Dental Cosmos.*

